# Cell Adhesion and Its Endocytic Regulation in Cell Migration during Neural Development and Cancer Metastasis

**DOI:** 10.3390/ijms13044564

**Published:** 2012-04-11

**Authors:** Takeshi Kawauchi

**Affiliations:** 1 Precursory Research for Embryonic Science and Technology (PRESTO), Japan Science and Technology Agency (JST), Saitama 332-0012, Japan; E-Mail: takeshi-kawauchi@umin.ac.jp; Tel.: +81-3-5363-3743; Fax: +81-3-5379-1977; 2 Department of Anatomy, Keio University School of Medicine, 35 Shinanomachi, Shinjuku-ku, Tokyo 160-8582, Japan

**Keywords:** membrane trafficking, invasion, neuronal migration, Cdk5, Rab5, Rab11

## Abstract

Cell migration is a crucial event for tissue organization during development, and its dysregulation leads to several diseases, including cancer. Cells exhibit various types of migration, such as single mesenchymal or amoeboid migration, collective migration and scaffold cell-dependent migration. The migration properties are partly dictated by cell adhesion and its endocytic regulation. While an epithelial-mesenchymal transition (EMT)-mediated mesenchymal cell migration requires the endocytic recycling of integrin-mediated adhesions after the disruption of cell-cell adhesions, an amoeboid migration is not dependent on any adhesions to extracellular matrix (ECM) or neighboring cells. In contrast, a collective migration is mediated by both cell-cell and cell-ECM adhesions, and a scaffold cell-dependent migration is regulated by the endocytosis and recycling of cell-cell adhesion molecules. Although some invasive carcinoma cells exhibit an EMT-mediated mesenchymal or amoeboid migration, other cancer cells are known to maintain cadherin-based cell-cell adhesions and epithelial morphology during metastasis. On the other hand, a scaffold cell-dependent migration is mainly utilized by migrating neurons in normal developing brains. This review will summarize the structures of cell adhesions, including adherens junctions and focal adhesions, and discuss the regulatory mechanisms for the dynamic behavior of cell adhesions by endocytic pathways in cell migration in physiological and pathological conditions, focusing particularly on neural development and cancer metastasis.

## 1. Introduction

**Figure 1:**
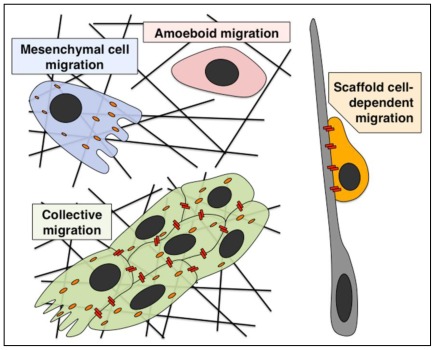

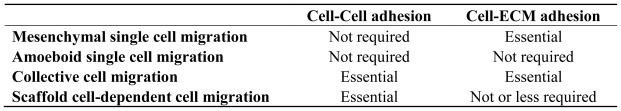
Classification of cell migration types. Orange dots: cell-ECM adhesions, Red rectangles: cell-cell adhesions, Black bars: ECMs. See Introduction.

Cell adhesion is essential for the organization and various biological functions of multicellular organisms. There are two major types of cell adhesions; cell-to-cell and cell-to-extracellular matrix (ECM), both of which consist of transmembrane cell adhesion molecules, intracellular scaffold or signaling proteins and cytoskeletons (Figure 1). Cadherin family cell adhesion molecules and their associated scaffold proteins, catenins, play important roles in the formation and functions of cell-cell adhesions, adherens junctions and desmosomes [1]. In contrast, the major transmembrane proteins at cell-ECM adhesions are integrin heterodimers [2]. These cell-cell and cell-ECM adhesion complexes are stabilized by actin cytoskeleton or intermediate filaments, but dynamically rearranged under some circumstances, such as cell migration and cancer metastasis [3,4]. In the initial step of cancer metastasis, tumor cells sometimes undergo epithelial-mesenchymal transition (EMT), which requires the disruption of cell-cell adhesions. Subsequently, the mesenchymal cancer cells begin migration and invade into the basement membranes and surrounding stromal tissues. The migrating mesenchymal cells constantly form new adhesions to ECM at the cell front, whereas a portion of the cell-ECM adhesions are disrupted by endocytosis at the rear. In contrast, recent reports indicate that some cancer cells maintain cell-cell adhesions and epithelial morphology during metastasis. This type of migration, called a collective cell migration, requires both cell-cell and cell-ECM adhesions. Furthermore, other cancer cells migrate in the absence of formation of any adhesions to neighboring cells or ECMs (an amoeboid migration). Thus, cancer cells exhibit various types of migration, which can be classified according to the cell adhesion properties [5] (Figure 1).

In addition to the various migration patterns of cancer cells, there is another type of migration, a scaffold cell-dependent migration, which only depends on cell-cell adhesions (Figure 1). This type of migration is rarely observed in cancer metastasis but occurs mainly in normal brain development. During the development of the cerebral cortex, immature neurons exhibit multi-step migration to form a functional brain [6]. The major migration mode of neurons is a radial glial cell-dependent migration, which is mediated by the endocytic recycling of cadherins, rather than integrins [7]. In this review, I summarize the structures of cell adhesions and their dynamic regulation by endocytosis and membrane trafficking. I also introduce the physiological and pathological roles of the regulation of cell adhesions in various types of cell migration in neural development and cancer metastasis.

## 2. Structures and Functions of Cell Adhesion Complexes

### 2.1. Adherens Junctions

**Figure 2:**
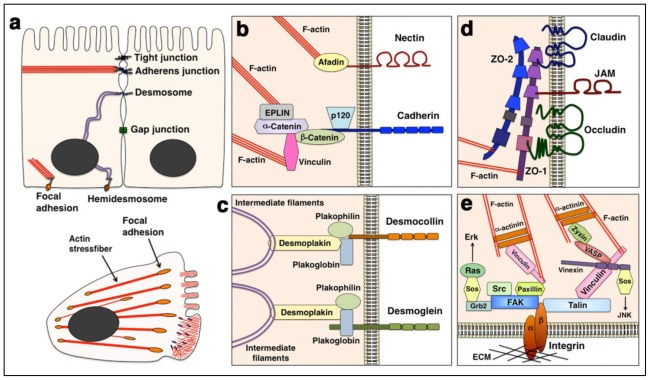
Molecular structures of cell-cell and cell-ECM junctions. (**a**) Epithelial cells contain both cell-cell junctions (Tight junctions, Adherens junctions, Desmosomes and Gap junctions) and cell-ECM junctions (Focal adhesions and Hemidesmosomes). While fibroblasts form cadherin-based cell-cell junctions, a major adhesion in fibroblasts is integrin-based focal adhesions. Red bars: actin filaments, Purple lines: intermediate filaments, Orange dots in the lower panel: focal adhesions, Purple dots in the lower panel; focal complex (immature focal adhesion); (**b–e**) Molecular components of adherens junctions (**b**), desmosomes (**c**), tight junctions (**d**) and focal adhesions (**e**).

Adherens junctions are observed in many cell types, including epithelial cells and fibroblasts (Figure 2a). The adherens junctions are required for the formation and maintenance of physical association of cells in a calcium-dependent manner [1]. Cadherin is a key molecule in calcium-dependent cell adhesion, whereas nectins and nectin-like proteins, which are calcium-independent cell adhesion molecules, also have important roles in adherens junctions; nectin and its associated intracellular molecule, afadin, initiate the formation of adherens junctions by the recruitment of a cadherin-catenin complex to the adherens junctions [8,9] (Figure 2b). While a cadherin superfamily consists of more than a hundred members, classic cadherins, including E-cadherin (cdh1) and N-cadherin (cdh2), comprise about 20 molecules. The extracellular domains of classic cadherins provide trans-homophilic binding to other cadherins on neighboring cells, and their intracellular domains directly interact with β-catenin and p120-catenin (p120^ctn^) [1,10]. α-catenin binds both β-catenin and actin filaments (F-actin), suggesting that β- and α-catenins link cadherin with actin cytoskeleton. However, it has been reported that the α-catenin, complexed with β-catenin and cadherin, rarely interacts with F-actin [11,12]. α-catenin binds to several actin-binding proteins, such as vinculin and EPLIN, and EPLIN concurrently forms a complex with cadherin, α-catenin, β-catenin and F-actin, suggesting that EPLIN and some other actin-binding proteins regulate the interaction between cadherins and F-actin cooperatively with α- and β-catenins [13,14] (Figure 2b).

### 2.2. Desmosomes

Desmosomes are patch-like intercellular junctions, and abundantly observed in skin and myocardium, both of which bear mechanical stress (Figure 2a). The desmosomes are composed of non-classic cadherins, desmogleins and desmocollins, and provide a strong intercellular connection [15,16]. Desmoglein and desmocollin are directly or indirectly associated with plakoglobin, plakophilin, desmoplakin and intermediate filaments (Figure 2c). Four and three isoforms of desmogleins and desmocollins have been identified in humans, respectively. Mutations or abnormal expression of desmosomal proteins are associated with several diseases, including arrhythmogenic right ventricular cardiomyopathy (ARVC), skin diseases and cancer [17–19]. Mutations in desmoglein-2, desmocollin-2, desmoplakin and plakoglobin have been identified in ARVC patients, whereas *N*-terminal deletion in desmoglein-1 leads to striate palmoplantar keratoderma [20,21]. In addition, desmogleins are targets of autoimmune diseases, pemphigus foliaceus and pemphigus vulgaris [17,19,22].

### 2.3. Tight Junctions and Gap Junctions

Tight junctions and gap junctions are cadherin-independent adhesions, although some crosstalk has been reported between cadherins and the components of tight junctions or gap junctions. Tight junctions are located at the most apical part of the lateral membranes in epithelial and endothelial cells (Figure 2a). Two major roles of tight junctions are “barrier” and “fence” functions [23,24]. At the tight junction strands, composed of claudin family and occludin, the intercellular distance between two neighboring cells is nearly zero, so that the tight junction restricts paracellular diffusion of substances and ions. In addition to the selective paracellular barrier function, tight junctions separate the plasma membrane into apical and basolateral domains to confine the diffusion of transmembrane proteins and lipids to the specific membrane domain as a fence. Both claudins and occludin bind to intracellular PDZ domain-containing proteins, ZO-1 (Figure 2d). Junctional adhesion molecule (JAM) family proteins are also associated with ZO-1 and localized at tight junctions [25].

Gap junctions consist of homo- or hetero-hexamers of connexin (Cx) proteins, such as Cx43, Cx26 and Cx32. Connexin hexamers, called connexons, form intercellular channels, which allow the diffusion of small molecules and electrical signals [26]. Some connexins are reported to interact with ZO-1, and Cx43 can be co-immunoprecipitated with N-cadherin and its associated catenins, suggesting that there is crosstalk between gap junctions and other junctional complexes [26,27]. Furthermore, components of gap junctions and tight junctions as well as cadherin-based adhesions have been associated with cancer [28–31].

### 2.4. Focal Adhesions

In addition to these cell-cell adhesions, cells also attach to ECMs, such as fibronectin, laminin, collagen and vitronectin. A major receptor for ECM is integrins. In humans, 18 α and 8 β subunits form at least 24 α–β heterodimers of integrins [2,32–36] (Table 1). Integrin heterodimers are localized at focal adhesions or hemidesmosomes, which are F-actin- or intermediate filament-based cell-ECM adhesion sites, respectively (Figure 2a), although some integrins serve cell-cell adhesion (Table 1). It has been reported that many scaffold or signaling proteins, including talin, vinculin, zyxin, focal adhesion kinase (FAK), Src, paxillin and p130^cas^, are localized at focal adhesions [37–39] (Figure 2e). Talin directly binds to β1-integrin and an actin-binding protein, vinculin. In addition to the role linking integrins and F-actin, talin is also required for integrin activation [40,41]. The binding of talin induces the switching of integrins from inactive to active conformations. Therefore, talin plays important roles in both an inside-out activation of integrin and outside-in signal for the maturation of focal adhesion. A recent report shows that SHARPIN perturbs the recruitment of talin to integrins and thereby inhibits the integrin activation [42]. Vinculin exhibits an intra-molecular interaction, which inactivates the scaffold function. In contrast, an active open form of vinculin interacts with many molecules, such as α-actinin, paxillin, vinexin and vasodilator-stimulated phosphoprotein (VASP) [43,44] (Figure 2e). While α-actinin, as well as vinculin, directly binds to F-actin, paxillin and vinexin interact with FAK and Sos, an activator for Ras, respectively [37,45,46]. FAK forms a complex with a Src kinase and Grb2, an adaptor protein that activates the Ras-MAP kinase pathway. Vinexin is reported to promote the activation of c-jun *N*-terminal kinase (JNK) [47]. Thus, focal adhesions function as not only a cell-ECM adhesion but also a signaling center to regulate cell migration, morphological changes, survival and proliferation.

## 3. Intracellular Trafficking Pathways

**Figure 3:**
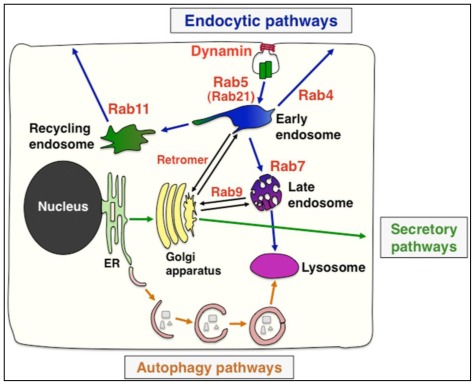
Intracellular trafficking pathways. Three major trafficking pathways are endocytic pathways (blue arrows), secretory pathways (green arrows) and autophagy pathways (orange arrows). Some cargo molecules are transported from endosomes to trans-Golgi network via retrograde pathways.

Membrane trafficking pathways are roughly classified according to their origins; secretory pathways originated from ER-Golgi, endocytic pathways from plasma membranes and autophagy pathways from cytoplasm [48–52] (Figure 3). Newly synthesized cell adhesion molecules are transported into the plasma membrane via secretory pathways. A recent report shows that two desmosomal cadherins, desmoglein-2 and desmocollin-2, are transported by the use of different plus-end motor proteins, kinesin-1 and kinesin-2, respectively [53]. Furthermore, the endocytic recycling and tight junction targeting of claudin and occludin are dependent on Rab13, whereas the transport of E-cadherin to the adherens junctions requires Rab8 in epithelial cell lines [54,55]. Therefore, the trafficking of cell adhesion molecules in distinct adhesion complex utilizes different transport machinery.

Cell adhesion molecules, engaged in adhesion complexes, are relatively stable, but they also undergo constant or stimuli-mediated remodeling by endocytosis and the recycling system. Endocytosis can be classified into several types, including clathrin-mediated endocytosis, caveolin-mediated endocytosis and macropinocytosis [56]. Endocytosis and its downstream endocytic pathways are mainly regulated by Rab family small GTPases [52,57–59] (Figure 3). Many types of endocytosis are mediated by the activities of dynamin, an atypical GTPase, and Rab5. Rab5 and its subfamily proteins, such as Rab21, Rab22 and Rab31, promote the internalization of transmembrane proteins and their transport into early endosomes. As early endosomes are also called “sorting endosomes”, there are many trafficking pathways from early endosomes; recycling pathway to plasma membrane, degradation pathway to lysosomes and retrograde transport to trans-Golgi network (TGN) (Figure 3). Whereas direct and indirect recycling pathways require Rab4 and Rab11, respectively, the lysosomal degradation pathway is dependent on Rab7. In contrast, the retrograde transports from early and late endosomes are regulated by the retromer complex and Rab9, respectively.

## 4. Cadherin Endocytosis and Epithelial-to-Mesenchymal Transition

**Figure 4:**
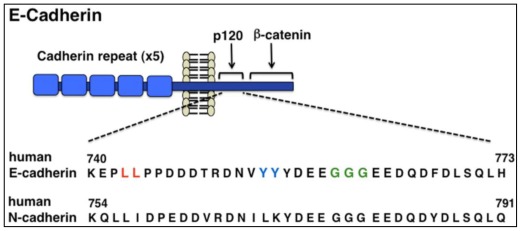
Dileucine and NVYYY motifs in E-cadherin intracellular region. A dileucine motif (shown in red characters) binds to endocytic adaptor proteins, such as AP-2, whereas a NVYYY motif (shown in blue characters), including the tyrosine phosphorylation site by a Src kinase, are required for a strong binding to p120^ctn^. A triple glycine (GGG) motif is also involved in the strong and static binding to p120^ctn^.

The cytoplasmic domain of E-cadherin contains a dileucine motif followed by a NVYYY motif at the membrane-proximal region and a β-catenin-binding site at the membrane-distal region (Figure 4). A dileucine motif is a binding site for clathrin adaptor complexes, such as AP-2, [60] and mutation in the dileucine motif in E-cadherin inhibits its endocytosis [61]. The dileucine motif in E-cadherin also binds to p120^ctn^, which masks the dileucine motif to prevent the endocytosis of E-cadherin [62–64]. Knockdown of p120^ctn^ results in the internalization of cadherins and loss of cell-cell contacts. However, the binding of Numb, a cytoplasmic adaptor, to p120^ctn^ cancels the p120^ctn^-mediated suppression of E-cadherin endocytosis, because Numb recruits an AP-2 clathrin adaptor complex [65]. Numb also interacts with the NVYYY motif on E-cadherin, which may prevent the binding of p120^ctn^ to Ecadherin [66]. Thus, Numb promotes E-cadherin endocytosis through two different mechanisms.

A recent structural and NMR study shows that the dileucine motif in E-cadherin serves a moderate and dynamic interaction with p120^ctn^, whereas the NVYYY motif, including the tyrosine phosphorylation site by a Src kinase, are required for a strong and static binding to p120^ctn^ [67]. Src-mediated phosphorylation of the NVYYY motif on E-cadherin induces the dissociation of p120^ctn^ and recruits a c-Cbl-related E3 ubiquitin ligase, Hakai. Hakai induces the ubiquitination of E-cadherin and subsequently its endocytosis and degradation [68]. In addition, Src and receptor tyrosine kinases, such as epidermal growth factor (EGF) receptor and brain-derived neurotrophic factor (BDNF) receptor, phosphorylate Tyr654 on β-catenin, resulting in the disruption of the cadherin β-catenin complex. In contrast, a cytoplasmic kinase, Fer, and hepatocyte growth factor (HGF) receptor phosphorylate Tyr142 on β-catenin, leading to the perturbation of the interaction between β-catenin and α-catenin [69,70].

Increased tyrosine phosphorylation occurs in growth factor-treated cells or oncogenic environments. Various growth factors, such as transforming growth factor β(TGFβ), HGF, fibroblast growth factor (FGF) and EGF, and oncogenic v-Src are known to induce an epithelial-mesenchymal transition (EMT) [71–73]. EMT progression involves the reduction in cell-cell adhesion, apico-basal polarity and epithelial markers, including E-cadherin, and the upregulation of mesenchymal markers, including N-cadherin, vimentin and α-smooth muscle actin. Although transcriptional regulation has crucial roles in the induction of EMT [74], the tyrosine phosphorylation of E-cadherin and catenins are also important events for EMT [75]. HGF treatment or v-Src expression activates Arf6, an Arf family small GTPase, which enhances E-cadherin endocytosis partly through the recruitment of Nm23-H1, an activator for dynamin [76,77]. HGF also induces Ras-mediated activation of RIN2, an activator for Rab5, increasing Rab5-dependent endocytosis of E-cadherin [78].

In addition to the clathrin-mediated endocytosis, caveolin-mediated endocytosis and macropinocytosis are involved in E-cadherin internalization [79,80]. EGF treatment promotes E-cadherin macropinocytosis into endosomes, in which E-cadherin still colocalizes with p120^ctn^, in MCF-7 cells [81]. Interestingly, E-cadherin and N-cadherin enhance macropinocytosis [82], implicating the positive feedback loop.

## 5. Integrin Endocytosis and Cell Migration

Integrin endocytosis and recycling are known to regulate the migration of many cell types, including neutrophils, fibroblasts and several cancer cell lines [83–85]. The endocytosis of integrins promotes the disassembly of focal adhesion and detachment from ECM at the rear of the cell, and thereby directs cell migration [86].

β5-integrin, which forms a heterodimer only with αv-integrin as a vitronectin receptor (Table 1), is observed at clathrin-coated membrane domains in cultured myotubes [87]. Furthermore, Numb, an endocytic adaptor for clathrin-mediated endocytosis, binds to β1-integrin, but not intracellular focal adhesion components, such as talin, vinculin, paxillin and FAK, and enhances integrin endocytosis and cell migration on fibrocentin or collagen [88]. Dab2 and autosomal recessive hypercholesteremia (ARH), another endocytic adaptor proteins, are also involved in integrin endocytosis and cell migration [89,90]. Suppression of Dab2 increases the cell surface levels of β1-, α1-, α2-, α3-, but not α5- or αv-integrins. Considering the binding specificity of integrins to ECM proteins (Table 1), Dab2 prefers to control the endocytosis of receptors for collagen and laminin, rather than fibronectin and vitronectin. Interestingly, Dab2-mediated endocytosis occurs evenly over the cell surface, suggesting that Dab2 increases the intracellular integrin pool that is recycled to create new focal adhesions [89].

Rab family small GTPases and Arf6 are known to regulate integrin endocytosis and intracellular trafficking [85,91]. Rab21 and Rab5 are co-immunoprecipitated with β1-integrin and control its endosomal trafficking in MDA-MB-231 breast cancer cells [92]. Internalized integrins are recycled back to the plasma membrane through Rab4-dependent direct recycling pathway or Rab11-dependent indirect recycling pathway via recycling endosomes (Figure 3). While phosphatidylinositol 3-kinase (PI3K) and Akt regulate the trafficking of αvβ3- and α5β1-integrins from Rab11-positive recycling endosomes to the plasma membranes, platelet-derived growth factor (PDGF) treatment promotes the Rab4-dependent fast recycling of αvβ3-integrin from early endosomes without the involvement of Rab11 [93,94]. Interestingly, β1-integrin also enhances PDGF receptor trafficking and directed cell migration [95].

Whereas a major fibronectin receptor is α5β1-integrin (Table 1), syndecan-4, a membrane-intercalated heparan sulfate proteoglycan, acts as a co-receptor for fibronectin [96]. A recent study shows that the binding of fibronectin to syndecan-4 induces α5β1-integrin endocytosis in a dynamin- and caveolin-dependent manner [97]. Syndecan-4 is associated with protein kinase Cα (PKCα), and this interaction is required for RhoG-regulated α5β1-integrin endocytosis and cell migration during wound healing. PKCα is also reported to interact with β1-integrin and to promote its endocytosis [98]. A portion of the internalized β1-integrin is recycled to the plasma membrane via PKCε activity, which promotes cell migration [99,100]. Thus, integrin trafficking, regulated by Rab family small GTPases and several kinases, has important roles in cell migration.

Integrin endocytosis is also required for the internalization of ECM proteins, such as vitronectin and fibronectin. In human skin fibroblasts, β5-integrin and vitronectin are co-localized at intracellular vesicles and transported into lysosomes [101]. Another report shows that caveolin-mediated endocytosis of β1-integrin is involved in the turnover of fibronectin [102]. The engagement of fibronectin causes the ubiquitination of α5-integrin, which is transported into lysosomes via the ESCRT pathway [103]. Interestingly, this selective sorting and degradation of fibronectin-bound α5β1-integrin is required for fibroblast migration [103].

Many reports have indicated that integrin trafficking is associated with cancer metastasis [91,104]. Increased expression of αvβ6-integrin is found in many cancers, including oral carcinoma. HS1-associated protein X-1 (HAX-1) directly binds to the cytoplasmic domain of β6-integrin and this interaction enhances the migration and invasion of oral squamous cell carcinoma cell lines through promotion of the clathrin-mediated endocytosis of αvβ6-integrin [105]. In addition, enhanced expression of Arf6 is observed in breast cancer cells, and GEP100 (also known as BRAG2), an activator for Arf6, is responsible for the invasive activity of MDA-MB-231 breast cancer cells [106]. Since it is known that GEP100 regulates the endocytosis of β1-integrin [107], Arf6-mediated trafficking of β1-integrin may contribute to the acquisition of invasive phenotypes in breast cancer cells. Furthermore, recent reports have revealed that oncogenic mutant forms of p53, a major tumor suppressor, enhance Rab11-dependent recycling of α5β1-integrin to promote invasive migration [108,109]. These observations strongly suggest that integrin trafficking is closely associated with cancer cell invasion and metastasis.

## 6. Roles of Cell Adhesion Dynamics in Cancer

### 6.1. Cancer Metastasis

Precise regulation of cell adhesion is important for various physiological events, such as normal development, immune system and wound healing, and its dysregulation is associated with many pathological states, including cancer metastasis.

**Figure 5:**
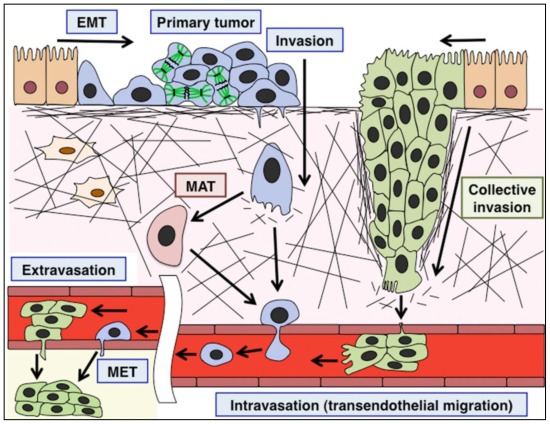
EMT-mediated invasion and collective invasion in cancer metastasis. Normal epithelial cells (orange cells) undergo EMT and form a primary tumor (blue cells). Some primary tumor cells invade and migrate into blood circulation as a multicellular strand (green cells). EMT: epithelial-mesenchymal transition, MAT: mesenchymal-amoeboid transition, MET: mesenchymal-epithelial transition.

The metastasis of carcinomas consists of several steps [73,110] (Figure 5). (i) Following the formation of a primary tumor, some tumor cells detach from the primary tumor; (ii) These tumor cells invade the basement membranes and local mesenchymal tissues; (iii) They undergo transendothelial migration into blood or lymphatic vessels (intravasation); (iv) Escaping from the immune system, they survive in the circulation; (v) The cancer cells attach to the vessel wall in the distant organ and migrate out of the vessel (extravasation); (vi) They proliferate to establish colonies at the secondary sites. These complicated processes require the dynamic regulation of cell adhesions through endocytic machinery as well as transcriptional regulation.

### 6.2. EMT in Cancer

Several studies pointed out the significance of EMT in cancer invasion [73,111,112], although it remains controversial because many metastatic tumors still retain epithelial properties and histopathological similarities to the primary tumors [113,114]. Furthermore, it is also unclear when metastasis comes into existence during tumor progression. A classical belief is that the invasion and subsequent dissemination of cancer cells occur after primary tumors have considerably expanded and have acquired high-motility properties through EMT. However, recent accumulating evidence indicates that the metastatic spread of tumor cells is an early step of cancer progression [115,116]. The disseminated tumor cells from HER-2 transgenic mammary glands are observed in lymph nodes or bone marrow at the premalignant phase, and early-disseminated cancer cells have the competency to induce lethal carcinosis [117]. Furthermore, there is no association between the tumor stage of human breast cancer and the number of disseminated cells in the bone marrow.

Regardless of whether it is an early or late event in cancer progression, the invasive migration of tumor cells is an initial important step for cancer metastasis. Although EMT has a limited role in cancer metastasis, many reports indicate that complete or incomplete EMT is associated with the metastasis of tumors, including breast cancer, hepatocellular carcinoma, colorectal cancer, pancreatic cancer and prostate cancer [118–124] (Figure 5). As described above, EMT is accompanied with downregulation of E-cadherin and upregulation of N-cadherin, the so-called “cadherin switch”. Transcriptional repression of E-cadherin is mediated by transcriptional repressors, snail family zinc finger proteins, Snail (SNAI1) and Slug (SNAI2), ZEB family transcriptional factors, ZEB1 and ZEB2 (also known SIP1), and basic helix-loop-helix proteins, Twist1 and Twist2. Snail and Slug also suppress the transcription of occludin and claudin-1, whereas ZEB2 targets Cx26 and plakophilin-2 [74].

TGFβ, a major EMT inducer, upregulates Snail and ZEB2 [125–127]. Furthermore, p12 (also known Cdk2ap), a downstream effector of TGFβ, induces EMT through Twist2 upregulation [128]. In addition to transcriptional regulators, the type II receptor of TGFβ phosphorylates Par6, a polarity protein, and the phosphorylated Par6 recruits an E3 ubiquitin ligase, Smurf1, leading to the degradation of RhoA [129]. Also, cooperatively with Raf-1, TGFβ promotes the endocytosis and lysosomal degradation of E-cadherin [130]. Therefore, TGFβ signaling is integral in EMT through the regulation of transcription, RhoA-dependent cytoskeletal remodeling and endocytosis, even though the downstream effect of TGFβ is cell type-specific [114].

It has been reported that microRNAs and alternative splicing regulators, ESRP1 and ESRP2, are involved in tumor cell migration and EMT [122,131–135]. A microRNA, miR-9 directly targets E-cadherin mRNA, resulting in an increase of cell migration and invasiveness [131,136]. Consistently, miR-9 is upregulated in breast cancer cells. In contrast, miR-200 and its related microRNAs, repress ZEB1, leading to the inhibition of EMT [122,132,133]. Interestingly, recent studies indicate that EMT is associated with the maintenance of stem cell properties, and the miR-200 family suppresses the “stemness” through the repression of Bmi1, KLF4 as well as ZEB1, suggesting that EMT may induce cancer stem cells [122,137–139].

### 6.3. Collective Invasion

In several cancers, including melanoma, breast cancer and colorectal cancer, cancer cells collectively migrate and invade other tissues without incurring EMT [113,140–143] (Figure 5). Collective migrating cells form multicellular strands (epithelial sheets) or coherent clusters detached from the primary tumors [5]. In either case, their cell-cell and cell-ECM adhesions are maintained, so that they can interact with neighboring cells and ECM during migration (Figure 1). In the collective migration of L-10 human rectal adenocarcinoma cells, HT-1080 fibroblastoma cells and MDA-MB-231 breast cancer cells, a leading cell (or a tip cell) in the front expresses MT1-MMP, a membrane-type matrix metalloproteinase, while the following cells at the back do not [142,144]. The activity of MT1-MMP is required for the transition from individual to collective cell invasion. Furthermore, in the co-cultures of carcinoma cells and stromal fibroblasts, the leading fibroblasts invade in an MMP-dependent manner and form tracks through α5-integrin-mediated RhoA activation and subsequent activation of myosin light chain (MLC), whereas carcinoma cells follow the tracks generated by the leading fibroblasts via Cdc42-mediated regulation of MLC [145]. These findings suggest that during collective cell migration, the leading and the following cells exhibit different properties and that the multicellular coordination of cell polarity and cytoskeletal regulation has important roles.

At the cell-cell adhesions in collective migrating cells, actomyosin contractility is kept at low levels to maintain cell-cell cohesion [146,147]. Discoidin domain receptor 1 (DDR1) interacts with polarity proteins, Par3 and Par6, which suppress Rho kinase/ROCK-mediated actomyosin contractility through the recruitment of RhoE [146]. Furthermore, DDR1 induces the upregulation of N-cadherin [148], and in collective migrating neural crest cells, N-cadherin inhibits membrane protrusion and Rac1 activity at cell-cell contacts [149,150]. Thus, cell adhesion molecules not only maintain cell-cell and cell-ECM contacts but also regulate intracellular signals to control collective cell migration.

Interestingly, a recent report has indicated the cooperative roles of EMT-mediated mesenchymal cells (EMT-cancer cells) and collective invading cells (non-EMT-cancer cells) [151,152]. EMT-cancer cells, but not non-EMT cancer cells, can invade and intravasate into the blood vessels, although they cannot establish metastasis in the distal organs. On the other hand, non-EMT-cancer cells can survive in the circulation and form the secondary tumors only if they are injected into the blood vessels, but not below the skin. When both EMT- and non-EMT cancer cells are inoculated subcutaneously, EMT cancer cells are located at the invasive front and the following non-EMT cancer cells can intravasate into the blood circulation. Furthermore, non-EMT cells extravasate and establish metastasis, suggesting that completing the entire metastatic process requires the cooperation between EMT- and non-EMT-cancer cells.

### 6.4. Amoeboid Migration

While TGFβ regulates the switch from collective to individual migration of breast cancer cells in a Smad-dependent manner [143], suppression of integrin causes the transition from collective to amoeboid migration. Amoeboid cell migration is independent of ECM attachment and protease activity; it requires actin cytoskeletal regulation to move forward [153] (Figures 1,5). It has been reported that the suppression of cofilin, an actin-binding protein, leads to the transition from amoeboid tumor cells to mesenchyme-like cells [154]. p27^kip1^, a cyclin-dependent kinase inhibitor protein that negatively regulates cell cycle progression, has also been reported to control mesenchymal-amoeboid transition (MAT) [155]. Interestingly, p27^kip1^ promotes cofilin-mediated actin reorganization as a downstream pathway of cyclin-dependent kinase 5 (Cdk5) in neurons [156]. Since Cdk5-mediated regulation of p27^kip1^ is associated with several cancers [157,158], a Cdk5-p27^kip1^ pathway might determine an amoeboid morphology through the regulation of cofilin in cancer.

## 7. Endocytic Regulation of Cell Adhesion in Neural Development

### 7.1. Cell Adhesion and Cerebral Cortical Development

Neural tissues, including cerebral cortex, are constructed from an epithelium-derived neural tube [159]. In the developing cerebral cortex, neural progenitors comprise a pseudostratified neuroepithelium (or ventricular zone), in which E- and N-cadherin-mediated adherens junctions and gap junctions, but not desmosomes, are observed [160–162]. ZO-1 is also localized at the apical parts of neuroepithelium, but tight junction structures are not observed after neural tube closure [161]. In the neuroepithelium, N-cadherin is transported by KIF3-mediated microtubule plus-end-directed motor complexes, composed of KIF3A, KIF3B and KAP3 [163], and knockout of N-cadherin or KAP3 results in the disruption of neuroepithelial structures [163,164]. Furthermore, N-cadherin-mediated adherens junctions may play an important role in proper neuronal differentiation because suppression of N-cadherin causes premature neuronal differentiation [165].

**Figure 6:**
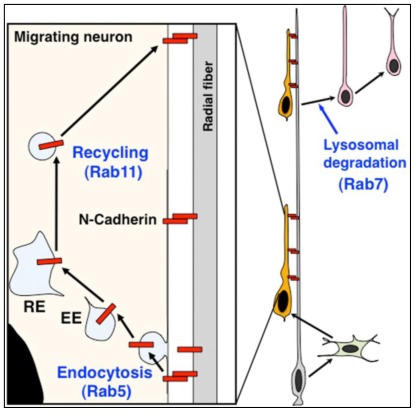
A scaffold cell-dependent migration in neural development. The migrating neurons in the developing cerebral cortex show radial glial fiber-dependent migration, which requires the endocytic trafficking of N-cadherin. EE: early endosomes, RE: recycling endosomes.

Differentiated immature neurons, generated from neural progenitors, migrate out of the neuroepithelium. These migrating immature neurons exhibit various morphological changes, and subsequently attach to radial glial fibers, pia-directed long processes derived from neural progenitors, in the developing cerebral cortex [6] (Figure 6). The morphological changes at the early phase of migration require the proper regulation of microtubule dynamics and actin cytoskeletal reorganization, mediated by Rac1-JNK-MAP1B and Cdk5-p27^kip1^-cofilin pathways, respectively [156,166–168]. Since p27^kip1^ controls the G1 length of neural progenitors and cell cycle exit through the inhibition of CDK activities [169,170], p27^kip1^ has dual functions in neural progenitors and migrating neurons, and therefore provides a molecular link between the cell cycle exit and migration start. Interestingly, mutations in ArfGEF2/Big2 cause periventricular heterotopia (caused by the migration defect in neurons) and microcephaly (caused by the proliferation defect in neural progenitors) in humans, suggesting that ArfGEF2/Big2 and its downstream Arf family small GTPases are also involved in both neural progenitor proliferation and migration start, likely through the regulation of membrane trafficking [171].

### 7.2. Scaffold Cell-Dependent Migration in Neural Development

After complex morphological changes, immature neurons begin a long-distance migration along radial glial fibers, called the locomotion mode of neuronal migration [172], which covers most of the neuronal migration route and is therefore a main contributor to neuronal migration and cortical layer formation [173]. This migration is a typical example of a scaffold cell-dependent migration (Figure 1). N-cadherin is required for the attachment of the locomoting neurons to the radial glial fibers [7,174] (Figure 6). A portion of this N-cadherin is internalized by a Rab5-dependent endocytosis and recycled to the plasma membrane via a Rab11-dependent indirect recycling pathway, suggesting that the intracellular trafficking of N-cadherin is essential for neuronal migration along radial glial fibers [7,52]. Although Rab5 is involved in not only clathrin-mediated endocytosis but also some caveolin-mediated endocytosis, it is known that at least clathrin function is required for cortical neuronal migration [175]. Recent reports reveal that a small GTPase, Rap1, regulates N-cadherin in migrating neurons as a downstream pathway of Reelin, whose mutation in mice results in the disruption of cortical layer structures, probably due to the neuronal migration defect [176,177]. In addition, Cx43 and Cx26, gap junction components are reported to regulate neuronal adhesion to radial glial fibers [178–180]. On the other hand, several reports indicate that β1-integrin is less important for the locomotion mode of migration [181,182], suggesting that a scaffold cell-dependent migration largely depends on cell-cell adhesions, but not cell-ECM adhesions (Figure 1).

Similar to the developing cerebral cortex in mice, N-cadherin is required for the migration of cerebellar granule neurons in zebrafish. This, however, is not a scaffold cell-dependent migration but a chain migration, an atypical mode of collective migration, which forms cell-cell contacts between migrating neurons [183]. In mice, cerebellar granule neurons exhibit a scaffold cell-dependent migration at the late phase of neuronal migration, and forms “interstitial junctions” and desmosome-like adhesions with Bergmann glial fibers [184]. A major component of interstitial junctions is astrotactin (ASTN1) [185]. Astrotactin is transported during neuronal migration and the trafficking is regulated by ASTN2 [186]. Thus, a scaffold cell-dependent migration requires the intracellular trafficking of cell-cell adhesion molecules, such as astrotactin and N-cadherin.

## 8. Conclusion Remarks

In physiological and pathological conditions, cell migration plays various important roles. Cell migration patterns are classified according to the cell adhesion properties (Figure 1). EMT-mediated single cell migration, amoeboid migration, collective migration and scaffold cell-dependent migration are differentially used during normal development and regeneration, suggesting that proper regulation of cell adhesion in migration is essential for tissue organization and maintenance. It is consistent with the fact that abnormal EMT and/or collective migration are closely associated with cancer metastasis. As described above, recent studies have uncovered the fact that endocytic trafficking is essentially required for several types of cell migration. However, research into the *in vivo* roles of endocytosis and intracellular membrane trafficking is just beginning. Better understanding of the regulatory mechanisms of membrane trafficking may uncover yet unknown critical roles in various biological fields, including developmental biology, neuroscience and cancer therapy.

## Figures and Tables

**Table 1:** Integrin subunits and their ligands.

Integrin subunits		Major Ligands
α1	β1	Collagen, Laminin
α2	β1	Collagen, Laminin, Tenascin,
α3	β1	Laminin, Collagen
α4	β1	Fibronectin, VCAM-1
	β7	MAdCAM-1, Fibronectin
α5	β1	Fibronectin
α6	β1	Laminin
	β4	Laminin
α7	β1	Laminin
α8	β1	Fibronectin, Tenascin
α9	β1	Tenascin
α10	β1	Collagen
α11	β1	Collagen
αIIb	β3	Fibronectin, Vitronectin, Fibrinogen
αv	β1	Vitronectin, Fibronectin
	β3	Vitronectin, Fibronectin, Fibrinogen
	β5	Vitronectin
	β6	Fibronectin, Tenascin
	β8	Vitronectin
αL	β2	ICAM-1, −2, −3
αM	β2	Fibrinogen, iC3b
αX	β2	Fibrinogen, iC3b
αD	β2	ICAM-3, VCAM-1
αE	β7	E-cadherin
